# Blood Storage Conditions Affect Hematological Analysis in Samples From Healthy Donkeys and Donkeys With Experimentally-Induced Endotoxemia

**DOI:** 10.3389/fvets.2020.00640

**Published:** 2020-09-10

**Authors:** Alejandro Perez-Ecija, Antonio Buzon-Cuevas, Raul Aguilera-Aguilera, Carlos A. Gonzalez-De Cara, Francisco J. Mendoza

**Affiliations:** ^1^Department of Animal Medicine and Surgery, University of Cordoba, Cordoba, Spain; ^2^Department of Pathology and Infectious Diseases, University of Surrey, Guildford, United Kingdom

**Keywords:** donkey blood, storage temperature, storage time, sepsis, hematologic analysis

## Abstract

Preanalytical factors such as storage time and temperature are proved to induce marked artifactual changes in hematological parameters in horses, small animals and humans. These errors can mirror findings typical of endotoxemia, leading to dangerous misdiagnosis. Since donkeys are common in warm climates and remote regions, blood samples from this species can be subjected to long lasting travels from the farm to the nearest laboratory, frequently under suboptimal conditions. Moreover, as other equids, donkeys are prone to suffer endotoxemia. Nonetheless, stability has not been evaluated in samples for hematology in this species. The aim of this study was to characterize the effect of temperature and storage time in hematological parameters from healthy donkeys and donkeys with induced endotoxemia. Blood samples were collected from six healthy female Andalusian donkeys and stored for 6, 12, 24, and 48 h at several temperatures (4, 24, and 35°C). Endotoxemia was induced in the same animals by an intravenous LPS infusion and samples obtained 30 min post-infusion were handled similarly. Hematological analysis was performed using a laser-based analyzer and blood smear examination. Storage at 24°C caused significant neutropenia after 48 h as well as morphological changes typical of endotoxemia in blood from healthy donkeys as soon as 24 h post-storage. Samples kept at 35°C displayed more profound and earlier artifactual variations. Conservation at 4°C did not cause any significant change in blood parameters. Prolonged (48 h) storage of samples from animals with induced endotoxemia at 24 and 35°C accentuated pre-existing leukopenia and neutropenia. These findings highlight that donkey samples should be stored at 4°C, instead of 24°C as recommended for horses. Moreover, blood smear interpretation should be cautious in samples stored for longer than 24 h and could be misleading when blood is kept at 35°C.

## Introduction

There is a growing interest worldwide in the use of donkeys as pet companion, in assisted therapy, and in high-quality alimentary by-products production, along with their traditional roles in agriculture and transport in developing countries (1–3). Accordingly, the caseload of donkeys referred to veterinary hospitals and the number of donkey blood samples delivered to laboratories have increased in the last years.

Numerous differences have been demonstrated between donkeys and horses (4–6), including marked discrepancies on reference ranges of hematological parameters using either impedance or laser-based analyzers (7–11). Thus, the extrapolation of reference ranges and laboratory guidelines between both species must be avoided.

Preanalytical factors such as storage time and temperature have been related to significant artifacts in hematological parameters in several species (12–17), leading to species-specific recommendations on sample handling. Since donkeys are commonly located in warm and remote regions (18), their blood samples are frequently subjected to long duration travels from the farm to the nearest laboratory, usually under inadequate cold-chain maintenance. Moreover, delays in hematological analysis can also occur due to sample collection on weekends or holidays, overwork in the laboratory, equipment breakdown, etc.

In addition, some of the artifactual changes caused by deficient storage in humans and dogs, such as pseudoleukocytopenia or pseudotoxic changes, can mimic typical features of the systemic inflammatory response syndrome (SIRS) or endotoxemia, leading to erroneous diagnosis (19, 20). Endotoxemia is assumed to be as common in donkeys as in horses (21), but little is known about the hematological response to this disturbance in this species (21, 22).

At the moment, data on the stability of donkeys' hematological parameters during storage are not available. Moreover, whether findings in blood from endotoxemic donkeys could be mimicked by storage artifacts is also unknown. Therefore, the aim of this work was to study the effect of temperature and storage duration on hematological parameters from healthy donkeys and donkeys with experimentally-induced endotoxemia. Our hypothesis was that conservation of donkey samples during large periods and at warm temperatures could cause abnormal findings similar to those observed during induced endotoxemia.

## Materials and Methods

### Animals

Six healthy adult (7.6 ± 0.8 years old) Andalusian non-pregnant jennies (348.3 ± 38.9 kg) housed in the facilities of the Veterinary Teaching Hospital of the University of Cordoba were included in this study. Animals had free access to drinking water and alfalfa hay and oat straw. Jennies were dewormed and had not received any treatment for at least 2 months prior to the study. Animals had no previous history of SIRS or endotoxemia-inducing diseases (e.g., colic, pleuropneumonia, diarrhea). Donkeys were healthy based on clinical history, physical examination and blood work profile (complete blood count, total protein, fibrinogen, albumin, aspartate transaminase -AST-, gamma-glutamyl transferase -γGT-, total bilirubin, creatinine and urea concentrations).

This study was carried out in accordance with the principles of the Basel Declaration and recommendations of the Royal Decree 1386/2018, which establishes the basic rules applicable for the protection of animals used in experimentation and other scientific purposes, Spanish Government. The study was conducted under permits 19-03-15-212 (Welfare Committee of the Conserjeria de Salud y Familias, Junta de Andalucia) and 2015PI/05 (Welfare Committee of the University of Cordoba).

### Experimental Design

Thirteen ml of blood was aseptically collected from the left jugular vein of each animal and carefully transferred to 1 ml K_3_EDTA tubes (Idexx VetCollect tubes, Idexx Europe, Hoofddorp, the Netherlands) (experiment A). One week later, endotoxemia was induced following previous protocols described in donkeys and horses (21, 23, 24). Briefly, a polyurethane catheter (Milacath, Mila International Inc., KY, USA) was aseptically placed in the left jugular vein, and 20 ng/kg of LPS (Escherichia coli O55:B5, Sigma-Aldrich Quimica, Madrid, Spain) diluted in 500 ml of sterile saline was administered over 30 min. Blood samples (13 ml) were collected 30 min post-LPS infusion into K_3_EDTA tubes (experiment B).

### Sample Handling and Measurements

One blood sample from experiments A and B was analyzed immediately after the collection (time 0 h). The rest of the tubes were divided in three groups (four tubes of 1 ml each one): one group was stored at 4°C in a refrigerator with an external thermostat controller (TSG505 refrigerator, ThermoFisher Scientist, Waltham, MA, United States), another at controlled room temperature (24°C, using the room air conditioning, a thermostat and a thermometer) and the last one was maintained in an incubator with an external thermostat controller at 35°C (Incubat, JP Selecta, Barcelona, Spain). One tube from each group was retrieved and, after homogenization, analyzed at the following times: 6, 12, 24, and 48 h post-collection using a laser-based hematological analyzer (LaserCyte, Idexx Europe, Hoofddorp, the Netherlands) with a previously validated donkey-specific setup (10). Samples stored at 4°C were raised to room temperature prior to analysis. All tubes were handled in a similar manner and aseptically in order to avoid bacterial contamination, mechanical damage, sample deterioration and any type of bias.

The following parameters were obtained: white blood cell (WBC) count, WBC differential counts, red blood cell (RBC) count, platelet (PLT) count, hematocrit (HCT), hemoglobin concentration, mean corpuscular volume (MCV), mean corpuscular hemoglobin (MCH), mean corpuscular hemoglobin concentration (MCHC), red cell distribution width (RDW), mean platelet volume (MPV) and platelet distribution width (PDW). Flags displayed by the analyzer were also recorded: VRL (value out of range), RD (RBC and/or platelets abnormal distribution), WD (WBC abnormal distribution) and PA (platelet aggregates).

Additionally, blood smears were prepared by triplicate from each sample. Blood films were air-dried and stained with a modified Wright–Giemsa method (Sigma-Aldrich, St. Louis, MO, United States). Two veterinarians with experience in clinical pathology blindly evaluated every blood smear using a light microscopy (CX31 microscope, Olympus CO., Tokyo, Japan) and ×40 and ×100 objectives. The following morphological abnormalities were recorded: % crenocytes, % ruptured WBC, platelet clumps, toxic neutrophils, pyknotic leukocytes and degranulated eosinophils. Percentages were calculated over a 400 WBC or RBC count. Grading of toxic neutrophils was adapted from a previous report in horses using a scale of 0–9 (25). Briefly, this scale is based on the severity of cytoplasmic vacuolation, cytoplasmic basophilia and Döhle bodies observed and the percentage of neutrophils affected. The rest of the abnormalities were classified as 0 = absent, 1 = scattered, 2 = moderate, 3 = frequent and 4 = highly frequent.

### Statistical Analysis

Normality was assessed by a Kolmogorov-Smirnov test. Every hematological analyte, % crenocytes and % ruptured WBC were normally distributed and these results were expressed as mean ± standard deviation of the mean (SD). The rest of morphological parameters (ordinal variables) were non-normally distributed and expressed as median and interquartile range (IQR: 25th percentile−75th percentile); with percentiles calculated using the Tukey's-Hinges test.

Collection time results were compared with post-storage data using an analysis of variance (ANOVA) of repeated measures followed by a Dunnett test. The effect of storage on ordinal variables was analyzed using a Friedman test followed by a Dunn's *post hoc* test. A *p*-value < 0.05 was considered significant. Inter-observer agreement for categorical variables in blood smears was adapted from a previous report (26). Agreement was calculated using the weighted Fleiss's kappa method with the following interpretation: < 0.20 no agreement; 0.21–0.39 minimal agreement; 0.40–0.59 weak agreement; 0.60–0.79 moderate agreement; 0.80–0.90 strong agreement; >0.90 almost perfect agreement. Statistical analysis was performed using a commercial statistical software (SPSS Statistics 24, IBM, Chicago, IL, USA).

## Results

### Experiment A: Effect of Storage Time and Temperature on Blood From Healthy Donkeys

Results are shown in Table 1. Storage at 35°C caused a significant (*p* < 0.05) reduction in neutrophils compared to basal, both at 24 and 48 h. When stored during 48 h, this temperature also caused significant (*p* < 0.05) decreased WBC, RBC, HCT and MCV. Conservation at 24°C also led to a significant (*p* < 0.05) neutropenia at 48 h. There were not significant changes in hematology parameters when blood was stored at 4°C.

**Table 1:** Effect of temperature and storage time on blood samples from healthy donkeys.

**Parameter**	**Collection**	**4**^**°**^**C**				**24**^**°**^**C**				**35**^**°**^**C**			
	**0 h**	**6 h**	**12 h**	**24 h**	**48 h**	**6 h**	**12 h**	**24 h**	**48 h**	**6 h**	**12 h**	**24 h**	**48 h**
WBC (× 10^3^/μl)	8.9 ± 2.1	9.5 ± 1.9	9.2 ± 2.8	9.3 ± 1.7	8.4 ± 2.1	9 ± 1.8	9.1 ± 2.2	9.5 ± 2.9	5.9 ± 2.1	9.1 ± 2.3	9.1 ± 2.0	6.0 ± 0.8	4.2 ± 1.4^a^
RBC (× 10^6^/μl)	6.3 ± 0.3	6.5 ± 0.4	6.2 ± 0.8	6.3 ± 0.2	6.1 ± 0.4	5.8 ± 0.4	5.2 ± 1.0	6.1 ± 0.4	4.9 ± 1.5	5.8 ± 0.3	5.6 ± 0.2	4.5 ± 1.7	2.3 ± 1.1^a^
Hb (g/dl)	12.5 ± 0.9	11.6 ± 0.9	13.0 ± 0.9	12.3 ± 0.8	13.0 ± 1.1	12.2 ± 0.8	12.1 ± 0.9	12.1 ± 0.9	12.2 ± 1.0	12.2 ± 0.8	12.7 ± 0.9	11.8 ± 1.0	13.0 ± 1.2
HCT (%)	35 ± 2.5	31 ± 3.1	34 ± 5.0	35 ± 2.4	34 ± 3.1	32 ± 3.4	31 ± 3.1	34 ± 2.3	28 ± 9.0	33 ± 2.1	33 ± 1.6	26 ± 10	17 ± 9.7^a^
MCV (fl)	56 ± 1.5	56 ± 1.0	56 ± 1.0	56.2 ± 1.6	56.2 ± 1.4	56 ± 1.2	55 ± 1.4	56.6 ± 1.4	56.8 ± 1.7	56 ± 0.9	56 ± 1.1	56.6 ± 2.0	49.6 ± 3.6^a^
MCH (pg)	20 ± 0.8	21 ± 0.3	21 ± 1.9	19.6 ± 0.7	21.2 ± 0.8	20 ± 0.8	23 ± 4.2	19.8 ± 1.3	28.4 ± 15	20 ± 0.9	21 ± 2.3	30.5 ± 16	75.2 ± 50
MCHC (g/dl)	35 ± 1.4	37 ± 8.4	37 ± 3.9	34 ± 6.3	37 ± 1.3	37 ± 2.0	40 ± 7.6	35 ± 1.4	50 ± 2.7	36 ± 9.0	37 ± 4.1	54 ± 2.9	95 ± 47
RDW (%)	19 ± 0.7	19 ± 0.4	19 ± 0.6	19 ± 0.5	19 ± 0.6	19 ± 0.6	19 ± 0.4	19 ± 0.6	18 ± 0.9	19 ± 0.7	19 ± 0.7	19 ± 1.0	20 ± 0.8
PLT (× 10^3^/μl)	398 ± 78	379 ± 62	316 ± 51	277 ± 61	272 ± 66	374 ± 87	311 ± 71	329 ± 67	372 ± 213	344 ± 88	307 ± 76	259 ± 90	496 ± 268
MPV (fl)	4.5 ± 0.4	5.0 ± 0.3	5.0 ± 0.5	5.1 ± 1.0	5.1 ± 0.8	3.9 ± 0.6	3.7 ± 0.5	4.5 ± 0.4	4.1 ± 0.4	4.4 ± 0.7	4.5 ± 0.6	4.0 ± 0.8	4.5 ± 0.3
PDW (%)	18 ± 0.4	19 ± 0.6	19 ± 0.4	20.0 ± 1.5	20.6 ± 1.0	18 ± 0.2	18 ± 0.4	18.8 ± 0.5	19.8 ± 0.8	19 ± 1.1	18 ± 0.3	20.0 ± 1.0	22.0 ± 1.2
Neu (× 10^3^/μl)	5.3 ± 1.9	5.3 ± 2.1	5.5 ± 2.3	5.3 ± 1.8	4.4 ± 2.1	4.9 ± 1.9	5.0 ± 1.9	5.0 ± 2.5	2.2 ± 1.2^a^	5.3 ± 2.0	4.7 ± 1.7	2.0 ± 1.2^a^	0.6 ± 0.5^a^
Lym (× 10^3^/μl)	2.5 ± 0.9	3.0 ± 1.1	3.0 ± 1.3	2.5 ± 0.4	2.3 ± 0.8	2.9 ± 1.1	2.8 ± 1.0	2.9 ± 0.8	2.4 ± 0.8	3.3 ± 0.9	2.9 ± 1.0	2.2 ± 0.3	2.0 ± 0.9
Mono (× 10^3^/μl)	0.4 ± 0.1	0.5 ± 0.1	0.4 ± 0.1	0.5 ± 0.1	0.6 ± 0.1	0.4 ± 0.1	0.5 ± 0.1	0.5 ± 0.2	0.5 ± 0.2	0.5 ± 0.1	0.6 ± 0.2	1.1 ± 0.8	0.9 ± 1.0
Eos (× 10^3^/μl)	0.5 ± 0.1	0.6 ± 0.1	0.7 ± 0.1	0.8 ± 0.1	0.8 ± 0.1	0.6 ± 0.1	0.7 ± 0.1	0.8 ± 0.1	0.6 ± 0.3	0.7 ± 0.1	0.6 ± 0.1	0.5 ± 0.2	0.6 ± 0.2
Baso (× 10^3^/μl)	0.04 ± 0.0	0.06 ± 0.0	0.06 ± 0.0	0.06 ± 0.0	0.06 ± 0.0	0.05 ± 0.0	0.04 ± 0.0	0.05 ± 0.0	0.04 ± 0.0	0.05 ± 0.0	0.06 ± 0.0	0.04 ± 0.0	0.03 ± 0.0

*Results are expressed as mean ± SD. WBC, white blood cell count; RBC, red blood cell count; Hb, hemoglobin concentration; HCT, hematocrit; MCV, mean corpuscular volume; MCH, mean corpuscular hemoglobin; MCHC, mean corpuscular hemoglobin concentration; RDW, red cell distribution width; PLT, platelet count; MPV, mean platelet volume; PDW, platelet distribution width; Neu, neutrophil count; Lym, lymphocyte count; Mono, monocyte count; Eos, eosinophil count; Baso, basophil count*.

a *p < 0.05 compared to Time 0 h (collection time) by a repeated-measures one-way ANOVA. n = 6*.

Fourteen samples (14 out of 78, 17.9%) were flagged as VRL (13 samples stored at 35°C and 1 at 24°C). The flags WD (6/78, 7.7%) and RD (5/78, 6.4%) were displayed only in samples at 35°C. Only samples stored for 48 h were flagged.

Morphological changes caused by storage in blood from healthy donkeys are displayed in Table 2A. Toxic neutrophils were increased (*p* < 0.05) in blood kept at 24 and 35°C for 24 or 48 h, but no changes were observed at 4°C. Crenocytes were significantly more common in every sample kept at 24 and 35°C, and samples stored at 4°C during 12, 24, and 48 h. Ruptured WBC were significantly more common in every sample kept at 35°C, and samples stored at 4 or 24°C during 12, 24, and 48 h. Pyknotic leukocytes significantly increased beginning at 12 h (35°C) and 24 h (4 and 24°C). Degranulated eosinophils significantly increased at 48 h at every temperature, as well as in samples kept during 24 h at 35°C.

**Table 2:** Effect of temperature and time storage on morphological findings in blood smears from healthy donkeys **(A)** and donkeys with experimentally-induced endotoxemia **(B)**.

**Artifact**	**4**^**°**^**C**					**24**^**°**^**C**				**35**^**°**^**C**			
	**0 h**	**6 h**	**12 h**	**24 h**	**48 h**	**6 h**	**12 h**	**24 h**	**48 h**	**6 h**	**12 h**	**24 h**	**48 h**
**(A)**													
% crenocytes	1 ± 0	3 ± 1	7 ± 1^a^	13 ± 3^a^	16 ± 2^a^	10 ± 1^a^	10 ± 3^a^	11 ± 3^a^	15 ± 3^a^	10 ± 2^a^	15 ± 4^a^	21 ± 4^a^	25 ± 3^a^
% ruptured WBC	1 ± 1	4 ± 1	9 ± 1^a^	17 ± 2^a^	24 ± 4^a^	3 ± 1	10 ± 1^a^	19 ± 3^a^	27 ± 5^a^	20 ± 5^a^	21 ± 6^a^	33 ± 9^a^	44 ± 9^a^
Toxic neutrophils	0 (0–0)	0 (0–0)	0 (0–1)	1 (0–1)	1 (0.25–1)	1 (0.25–1)	1 (0.25–1)	2.5 (2–3)^a^	3.5 (3–4)^a^	1 (0.25–1)	1 (0.25–1)	3.5 (3–4)^a^	4.5 (4–5)^a^
Pyknotic leukocytes	0 (0–0)	0 (0–0)	1 (0.25–1)	2 (2–2)^a^	2.5 (2–3)^a^	1 (0.25–1)	1 (0.25–1)	2.5 (2–3)^a^	2.5 (2–3)^a^	1 (0.25–1)	2.5 (2–3)^a^	3 (3–3)^a^	3.5 (3–4)^a^
Degranulated eosinophils	0 (0–0)	0 (0–0)	0 (0–0.75)	1.5 (1–2)	2.5 (2–3)^a^	0 (0–0)	0 (0–0.75)	1.5 (1–2)	2.5 (2–3)^a^	0 (0–0.75)	0 (0–0.75)	2.5 (2–3)^a^	3.5 (3–4)^a^
Platelet clumps	0 (0–0)	0 (0–0.75)	1 (1–1)	1 (1–2)	1 (1–2)	0 (0–0)	0 (0–0.75)	1 (1–1)	1 (1–2)	0 (0–0.75)	0 (0–0.75)	1 (1–1)	1 (1–2)
**(B)**													
% crenocytes	5 ± 1	10 ± 2	15 ± 3^a^	14 ± 3^a^	20 ± 3^a^	10 ± 3	12 ± 2^a^	12 ± 4^a^	19 ± 4^a^	20 ± 3^a^	16 ± 5^a^	17 ± 5^a^	16 ± 5^a^
% ruptured WBC	2 ± 1	26 ± 6^a^	35 ± 7^a^	39 ± 8^a^	49 ± 10^a^	29 ± 8^a^	35 ± 8^a^	48 ± 11^a^	51 ± 10^a^	35 ± 6^a^	46 ± 9^a^	48 ± 9^a^	55 ± 8^a^
Toxic neutrophils	3.5 (3–4)	3.5 (3–4)	3.5 (3–4)	3.5 (3–4)	3.5 (3–4)	3.5 (3–4)	3.5 (3–4)	3.5 (3–4)	3.5 (3–4)	3.5 (3–4)	3.5 (3–4)	4 (3–4)	4 (3–4)
Pyknotic leukocytes	0 (0–0)	0 (0–0)	0.5 (0–1)	1.5 (1–2)	2 (2–2)^a^	0 (0–0)	1 (1–1)	1.5 (1–2)	2 (2–2)^a^	1.5 (1–2)	2 (2–2)^a^	2.5 (2–3)^a^	3.5 (3–4)^a^
Degranulated eosinophils	0 (0–0)	1.5 (1–2)	1.5 (1–2)	1.5 (1–2)	2 (2–2)^a^	1.5 (1–2)	1.5 (1–2)	2 (2–2)^a^	1.5 (1–2)	2 (2–2)	1.5 (1–2)	1.5 (1–2)	2 (2–2)^a^
Platelet clumps	1 (1–1)	1 (1–2)	1 (1–2)	1 (1–1)	1.5 (1–2)	1 (1–1)	1 (1–1)	1 (1–1)	1 (1–2)	1 (1–1)	1 (1–1)	1 (1–1)	1 (1–2)

*Results are expressed as mean ± SD (% crenocytes and ruptured WBC) or median (25th percentile−75th percentile) for the rest of artifacts. Toxic neutrophils were categorized in a scale 0–9. Pyknotic leukocytes, degranulated eosinophils and platelet clumps were classified in a scale 0–4. WBC, white blood cell counts; C, Celsius degrees*.

a *p < 0.05 compared to Time 0 h (collection time) by a repeated-measures one-way ANOVA (% crenocytes and ruptured WBC) or p < 0.05 compared to collection time using a Friedman test (rest of artifacts). n = 6*.

### Experiment B: Effect of Storage Time and Temperature on Blood From Donkeys With Experimentally-Induced Endotoxemia

All donkeys safely completed the study and developed typical features of SIRS such as tachycardia, fever and leukopenia. In addition, neutropenia, lymphopenia, eosinopenia and monocytopenia were also observed. Clinical data are reported in a previous study (21).

Results are shown in Table 3. Storage of blood from endotoxemic donkeys during 48 h either at 24 or 35°C caused significant decreases (*p* < 0.05) in WBC and neutrophil counts compared to basal results. Samples at 35°C also showed significant differences (*p* < 0.05) in lymphocytes, HCT, MCV, MCH, MCHC and PDW at 48 h. No significant changes were found between basal blood parameters from endotoxemic donkeys and results of samples stored at 4°C.

**Table 3:** Effect of temperature and storage time on blood samples from donkeys with experimentally-induced endotoxemia.

**Parameter**	**Collection 0 h**	**4**^**°**^**C**				**24**^**°**^**C**				**35**^**°**^**C**			
		**6 h**	**12 h**	**24 h**	**48 h**	**6 h**	**12 h**	**24 h**	**48 h**	**6 h**	**12 h**	**24 h**	**48 h**
WBC (× 10^3^/μl)	1.9 ± 0.4	1.6 ± 0.4	1.4 ± 0.2	1.4 ± 0.4	1.4 ± 0.4	1.6 ± 0.3	1.6 ± 0.4	1.5 ± 0.4	1.0 ± 0.1^a^	1.6 ± 0.3	1.4 ± 0.2	1.4 ± 0.1	0.7 ± 0.0^a^
RBC (× 10^6^/μl)	6.7 ± 1.9	7.2 ± 1.2	7.4 ± 1.3	7.9 ± 1.5	7.1 ± 2.8	6.8 ± 1.7	6.6 ± 0.8	7.4 ± 1.5	5.9 ± 2.8	7.6 ± 0.6	7 ± 1.5	6.6 ± 1.8	4.2 ± 0.7
Hb (g/dl)	14.9 ± 1.5	14.6 ± 1.8	14.8 ± 2.0	15.4 ± 2.3	14.9 ± 2.6	14.6 ± 1.8	14.0 ± 1.5	14.8 ± 1.9	14.6 ± 2.3	14.7 ± 1.0	15.3 ± 0.8	14.4 ± 2.4	15.5 ± 1.0
HCT (%)	37 ± 11	40 ± 6.1	40 ± 5.2	43 ± 8.2	39 ± 15	37 ± 8.1	36 ± 4.2	40 ± 6.6	32 ± 14	41 ± 5.5	38 ± 8.1	37 ± 10	22 ± 4.6^a^
MCV (fl)	54 ± 2.3	55 ± 2.8	54 ± 2.8	55 ± 1.9	55 ± 2.0	55 ± 3.4	54 ± 2	54 ± 2.2	55 ± 2.0	54 ± 3	54 ± 2.2	56 ± 2.0	51 ± 2.7^a^
MCH (pg)	23 ± 7.4	19 ± 1.2	20 ± 1.3	19 ± 1.4	23 ± 10	22 ± 1.6	21 ± 1.1	20 ± 2.5	29 ± 13	19 ± 1.5	24 ± 2.2	22 ± 4.0	36 ± 6.5^a^
MCHC(g/dl)	43 ± 4.2	38 ± 1.0	38 ± 1.0	38 ± 1.5	42 ± 1.8	41 ± 1.1	38 ± 1.9	37 ± 3.5	52 ± 9.1	35 ± 2.6	43 ± 8.1	40 ± 7.6	71 ± 9.5^a^
RDW (%)	19 ± 0.1	19 ± 0.2	19 ± 0.3	19 ± 0.1	19 ± 0.3	19 ± 0.3	19 ± 0.2	19 ± 0.3	19 ± 0.4	19 ± 0.3	19 ± 0.4	20 ± 0.5	20 ± 0.7
PLT (× 10^3^/μl)	145 ± 31	133 ± 31	112 ± 22	103 ± 23	104 ± 30	160 ± 31	168 ± 31	139 ± 42	102 ± 48	126 ± 14	97 ± 22	125 ± 58	148 ± 71
MPV (fl)	6.0 ± 0.7	6.1 ± 0.9	5.9 ± 0.4	5.8 ± 0.8	5.5 ± 0.7	4.9 ± 0.4	4.7 ± 0.5	5.2 ± 1.0	5.6 ± 1.3	4.9 ± 0.5	5.3 ± 0.5	4.7 ± 0.5	4.7 ± 1.1
PDW (%)	19 ± 0.4	20 ± 0.7	20 ± 0.4	21 ± 1.6	21 ± 0.7	20 ± 0.5	20 ± 0.5	21 ± 1.3	22 ± 2.0	19 ± 0.5	21 ± 1.4	20 ± 1.1	24 ± 1.7^a^
Neu (× 10^3^/μl)	0.6 ± 0.2	0.7 ± 0.3	0.6 ± 0.2	0.6 ± 0.2	0.4 ± 0.2	0.7 ± 0.2	0.7 ± 0.2	0.6 ± 0.2	0.1 ± 0.0^a^	0.7 ± 0.2	0.5 ± 0.1	0.4 ± 0.1	0.1 ± 0.0^a^
Lym (× 10^3^/μl)	1.1 ± 0.5	0.6 ± 0.2	0.6 ± 0.1	0.6 ± 0.2	0.7 ± 0.5	0.6 ± 0.3	0.7 ± 0.2	0.7 ± 0.3	0.7 ± 0.1	0.7 ± 0.2	0.6 ± 0.2	0.7 ± 0.2	0.3 ± 0.0^a^
Mono (× 10^3^/μl)	0.05 ± 0.0	0.0 ± 0.0	0.1 ± 0.0	0.1 ± 0.0	0.1 ± 0.0	0.0 ± 0.0	0.1 ± 0.0	0.1 ± 0.0	0.1 ± 0.0	0.1 ± 0.0	0.1 ± 0.0	0.1 ± 0.0	0.0 ± 0.0
Eos (× 10^3^/μl)	0.0 ± 0.0	0.1 ± 0.0	0.1 ± 0.0	0.0 ± 0.0	0.0 ± 0.0	0.1 ± 0.0	0.1 ± 0.0	0.0 ± 0.0	0.0 ± 0.0	0.1 ± 0.0	0.1 ± 0.0	0.0 ± 0.0	0.0 ± 0.0
Baso (× 10^3^/μl)	0.0 ± 0.0	0.0 ± 0.0	0.0 ± 0.0	0.0 ± 0.0	0.0 ± 0.0	0.0 ± 0.0	0.0 ± 0.0	0.0 ± 0.0	0.0 ± 0.0	0.0 ± 0.0	0.0 ± 0.0	0.0 ± 0.0	0.0 ± 0.0

*Results are expressed as mean ± SD. WBC, white blood cell count; RBC, red blood cell count; Hb, hemoglobin concentration; HCT, hematocrit; MCV, mean corpuscular volume; MCH, mean corpuscular hemoglobin; MCHC, mean corpuscular hemoglobin concentration; RDW, red cell distribution width; PLT, platelet count; MPV, mean platelet volume; PDW, platelet distribution width; Neu, neutrophil count; Lym, lymphocyte count; Mono, monocyte count; Eos, eosinophil count; Baso, basophil count*.

a *p < 0.05 compared to Time 0 h (collection time) by a repeated-measures one-way ANOVA. n = 6*.

Sixteen samples (16/78, 20.5%) were flagged as VRL (14 kept at 35°C and 2 at 24°C); 8 (10.2%) as WD (seven stored at 35°C and 1 at 24°C) and three samples stored at 35°C (3.8%) as RD. Only samples stored for 48 h were flagged.

Morphological abnormalities are shown in Table 2B. Both toxic changes and platelet clumps were already present at collection time and neither of them were significantly affected by storage. % crenocytes and % ruptured WBC significantly increased (*p* < 0.05) during storage at every temperature. Pyknotic leukocytes only were significantly increased when blood was kept at 35°C during 12, 24, and 48 h as well as in samples kept at 4 and 24°C during 48 h. Degranulated eosinophils were also increased in blood stored during 24 h (24°C) and 48 h (4 and 35°C).

### Inter-observer Agreement in Blood Smears

Agreement for toxic neutrophils and platelet clumps was strong (0.88 and 0.81, respectively); while was moderate for degranulated eosinophils (0.71). Slightly lower agreements were detected in samples stored at higher temperatures during 48 h.

## Discussion

This study evaluated the stability of EDTA whole-blood samples from healthy donkeys and donkeys with experimentally-induced endotoxemia under different storage conditions. Main artifactual changes, both in healthy and endotoxemic donkeys, were observed when blood was kept at 24 and 35°C during 24 and 48 h, while results from blood samples at 4°C were closer to the analysis performed at collection time (0 h).

A previous systematical study on storage of hematological samples in horses recommended conservation at 24°C (13). In our study, blood from healthy donkeys stored at room temperature developed toxic changes in neutrophils as soon as 24 h and neutropenia 48 h post-storage. Since neutropenia and the presence of toxic neutrophils are key features for the diagnosis of sepsis/SIRS in adults and neonates (25, 27), these artifacts could lead to misdiagnosis and unnecessary treatments and economic losses. These discrepancies between donkeys and horses in the effect of temperature on blood parameters could be due to different viability of neutrophils, although variations amongst analyzers used in each study should also be considered (12, 15). The pseudotoxic changes observed on blood smears kept at 24°C also has been reported in dogs (20). Lysed WBCs significantly increased (compared to basal) at 12 h in samples kept at 4 and 24°C. Since this artifactual change can interfere with the impedance and laser-based techniques used by most hematology analyzers to classify leukocytes, a cautious interpretation of differential counts should be recommended on aged samples from donkeys under these conditions.

Storage of blood samples at 4°C is considered more suitable in humans, rodents and dogs (14, 15, 17, 28). This has also been occasionally advised for horses by some authors (29), although published data contradicts this statement (13). In our study, no changes were observed using the analyzer in samples kept at this temperature. However, morphological artifacts (crenocytes and ruptured leukocytes) significantly increased in blood stored for 12 or more hours, which underlines the importance of a rapid blood smear evaluation, even when the sample is kept in the refrigerator. Platelet clumping has been reported to appear earlier in samples kept at 4°C in several species (12, 17). In our study, clumps were observed in samples kept at every tested temperature without significant differences. Nonetheless, these samples were not flagged with PA by the analyzer. While the manufacturer provides no information in this subject, the requirement of a certain amount of aggregates or markedly large clumps in order to display this flag could be a feasible explanation. Newer analyzers, able to perform both impedance and optical-based platelet counts, have been proved to partially solve this problem (17).

Samples stored at 4°C showed no increase in toxic indicators for at least 48 h, which is markedly longer than reported in dogs, where pseudo-Döhle bodies and other toxic changes were apparent at 24 h (20).

Little information is available about the effect of keeping blood in warm environments on hematological parameters. Storage at 35°C caused important changes on both red and white cell counts as soon as 24 h post-sampling, likely secondary to cell lysis and leukoagglutination (15, 19). In contrast, these parameters were not altered in a previous report on pigs, cattle and goats blood stored at 30°C during 48 h (30). Whether these differences are due to species-specific idiosyncrasies or variations in analyzer technologies should be studied. Morphological artifacts on blood smears at 24 and 35°C resembled those from endotoxemic donkeys, which could have prompted a misdiagnosis in these samples. Since lysed WBCs were significantly increased as soon as 12 h post-sampling, the impact of these abnormalities on the results of the analyzer should also be considered.

LPS administration caused quantitative hematological findings and morphological toxic changes similar to those reported in horses with endotoxemia and SIRS (25, 27). Leukocytopenic samples are proved to be more prone to artifactual changes during storage compared to normal samples in humans (31). Alterations related to storage in endotoxemic blood were similar to those observed in healthy samples, with refrigeration avoiding any significant difference compared to basal results. Prolonged (48 h) storage of endotoxemic samples at 24 and 35°C accentuated pre-existing leukopenia and neutropenia. Although this finding could not affect the diagnosis, it could influence on the prognosis and the evaluation of the response to the treatment (25). Interestingly, toxic neutrophils did not significantly vary during the storage at 24 and 35°C, which could be due to lower percentage of viable neutrophils.

Artifactual changes also depends on the analyzer used for hematological evaluation (12, 15). As previously reported in healthy donkeys (10), proper cell type recognition can be problematic in LaserCyte and blood smears are recommended to verify the results. No sample from this study was flagged as IQA (Internal Quality Assurance Error), which secure that every determination met the analyzer's internal quality assurance checks. Flagging was more prevalent in samples stored for 48 h at higher temperatures; as well as in endotoxemic blood, where cell viability was already altered. Cell lysis, cell swelling and artifactual cytoplasmic changes could be responsible for most of these warnings. The most common observed flag was related to high MCHC, indicating most probably artifactual hemolysis. As stated, platelet aggregates were consistently observed even in unflagged samples, indicating the necessity of a proper blood smear examination in donkey samples, even in the absence of flags.

One limitation of this study is the low number of animal included. Similarly, our results depend on the hematological analyzer used and further studies should be needed in order to describe storage effect on donkey blood using more advanced hematological analyzers. A more delayed sampling post-LPS infusion could have allowed more pronounced abnormalities in blood smear. Finally, it could be of interest to study storage artifacts in blood from donkeys with naturally occurring endotoxemia or SIRS.

To the author's knowledge, this is the first study evaluating the effects of storage temperature and time on donkey hematological parameters both in healthy and those with experimentally-induced endotoxemia Our findings underline the importance of storing donkey blood samples at 4°C, instead of 24°C as recommended for horses, and perform the analysis within 12 h of collection. Moreover, due to the appearance of pseudotoxic changes, blood smear interpretation should be cautious in samples stored longer than 24 h at 24°C or warmer environments.

## Data Availability Statement

The raw data supporting the conclusions of this article will be made available by the authors, without undue reservation.

## Ethics Statement

The study was conducted under permits 19-03-15-212 (Welfare Committee of the Conserjeria de Salud y Familias, Junta de Andalucia) and 2015PI/05 (Welfare Committee of the University of Cordoba).

## Author Contributions

AP-E, AB-C, and FM contributed conception and design of the study. AB-C, RA-A, CG-D, and FM performed the experiments concerning induced endotoxemia. AP-E, FM, and CG-D performed hematological determinations. FM organized the database and performed the statistical analysis. AP-E wrote the first draft of the manuscript. All authors contributed to the article and approved the submitted version.

## Conflict of Interest

The authors declare that the research was conducted in the absence of any commercial or financial relationships that could be construed as a potential conflict of interest.
